# Workplace violence and body-worn camera use among emergency medical technicians in South Korea: a nationwide cross-sectional survey

**DOI:** 10.1038/s41598-026-57707-8

**Published:** 2026-06-12

**Authors:** Min-Ki Ban

**Affiliations:** National Fire Research Institute, 376 SongAk-ro, Asan City, 31555 Chungcheongnam-do Republic of Korea

**Keywords:** Emergency medical services, Workplace violence, Body-worn cameras, Paramedics, Occupational safety, South Korea, Prehospital care, Health care, Health occupations, Medical research, Risk factors

## Abstract

Emergency medical technicians (EMTs) frequently experience workplace violence in prehospital settings; however, evidence on the role of body-worn cameras (BWCs) in this context is limited. This study examined the factors associated with frequent workplace violence, the perceived need for BWCs, and actual BWC use among South Korean EMTs, incorporating individual- and regional-level indicators. A nationwide cross-sectional survey was conducted among EMTs employed by provincial fire departments in South Korea. Survey data on sociodemographic characteristics, work conditions, workplace violence over the past year, and BWC-related perceptions and behaviors were linked to province-level assault indicators from the National 119 emergency medical services (EMS) Statistical Yearbook (2020–2024). The main outcomes included frequent workplace violence, high perceived need for BWCs, and BWC use. Regional assault burden was modeled as tertiles of a five-year mean provincial assault index and as a continuous measure per 10-unit increase. All primary outcomes were self-reported within a knowledge–attitude–practice (KAP) survey design. Multivariable logistic regression was performed to estimate adjusted odds ratios (ORs) and 95% confidence intervals (CIs). To address within-province clustering, Generalized Estimating Equations (GEE) were additionally used as a sensitivity analysis. Of 3,595 EMTs included in the descriptive analyses, 2,555 provided complete violence data. Nearly all respondents (99.7%) reported some workplace violence in the past year, and 17.3% were classified as experiencing frequent workplace violence. Overall, 64.4% reported using a BWC, and 87.9% expressed a high perceived need. Frequent violence was associated with younger age, longer EMS career, higher daily call volume, and working as an EMT rather than an ambulance driver, but not with regional assault burden. High perceived need for BWCs was more common among female EMTs and varied by age, again without clear associations with regional assault indices. In contrast, actual BWC use was associated with job position, shorter EMS career, lower call volume, and high perceived need, and was inversely associated with higher regional assault burden. Workplace violence was highly prevalent among South Korean EMTs, and the perceived need for BWCs was widespread. Individual and work-related factors, rather than province-level assault burden, were the key determinants of frequent violence and BWC-related perceptions, whereas BWC use was less common in regions with higher recorded assault indices.

## Introduction

Emergency medical technicians (EMTs) frequently encounter verbal abuse, threats, and physical assault when providing care in prehospital settings^1–5^. Workplace violence against healthcare workers, including emergency medical services (EMS) providers, has been linked to psychological distress, burnout, reduced job satisfaction, and potential adverse effects on the quality and safety of patient care^1,2,4–7^. Despite growing recognition of this problem, empirical evidence on risk factors and effective preventive strategies remain limited, particularly in regions outside North America and Europe^1–5^.

Body-worn cameras (BWCs) have been adopted across several public safety sectors, most prominently in law enforcement, to deter aggression, document encounters, and enhance accountability^8–15^. Evidence from randomized trials and systematic reviews suggests that BWCs can reduce complaints and certain forms of violence against police officers, although observed effects vary by context and implementation^12,13^. In prehospital care, EMTs operate in patients’ homes and public spaces, often amid heightened emotional stress. In this setting, BWCs may offer protective benefits but also raise concerns regarding patient privacy, clinical decision-making, and provider–patient relationships^8–11,14,15^.

In South Korea, the national 119 EMS system delivers standardized ambulance services across all provinces^16^. National statistics report a substantial burden of assault-related incidents involving EMTs^16^, and several local EMS agencies have piloted or adopted BWCs as protective measures^8–11,14,15^. Despite these initiatives, nationwide evidence remains limited regarding the prevalence of violence against EMTs, their perceived need for BWCs, and the factors associated with actual camera use in routine practice, particularly within a unified EMS system^7^. Moreover, it remains unclear whether regional differences in assault burden translate into variation in EMTs’ individual experiences and behaviors, as this relationship has not been systematically evaluated.

To address this gap, we conducted a nationwide survey of 119 EMTs in South Korea and linked individual-level responses with provincial assault indicators derived from official EMS statistics^16^. Guided by a knowledge–attitude–practice (KAP) framework widely used to examine how perceptions translate into preventive behaviors^17,18^, and using multivariable logistic regression, we aimed to clarify the roles of individual characteristics, work conditions, and regional assault burden in (1) frequent exposure to workplace violence, (2) a high perceived need for BWCs, and (3) actual BWC use during EMS calls. Our findings are intended to inform EMS workforce protection policies and guide future evaluations of BWC implementation in prehospital care. Specifically, the study addressed three pre-specified research questions: RQ1: What individual-level and regional factors are associated with frequent workplace violence among South Korean 119 EMTs? RQ2: What factors are associated with a high perceived need for BWCs? RQ3: What factors are associated with actual BWC use during EMS calls, including individual characteristics, violence exposure, perceived need, and regional assault burden?

## Methods

### Study design and setting

We conducted a nationwide, cross-sectional analytical study using data from an online survey of 119 EMTs employed by provincial fire departments in South Korea. The survey was designed within a knowledge–attitude–practice (KAP) framework^17,18^, assessing workplace violence exposure (knowledge/experience), BWC-related perceptions and perceived need (attitude), and actual BWC use (practice). All three primary outcomes were self-reported by participants based on their personal experiences and perceptions during the study period. To account for regional variation in assault risk, individual survey responses were linked to province-level assault indicators obtained from the National 119 Emergency Medical Service Statistical Yearbook (2020–2024)^16^.

### Survey instrument

The questionnaire used in this study was adapted from previously validated instruments examining workplace violence and BWC-related perceptions among South Korean EMTs^19,20^. Specifically, it was based on a tool developed by Lim & Yang^19^, which refined and supplemented the questionnaire originally designed by Pyo^20^. These earlier instruments had been psychometrically evaluated in prior studies, supporting the reliability and content validity of the current survey. All items used a 5-point Likert scale, and responses were collected anonymously.

### Study population and recruitment

The target population included EMTs who regularly staffed 119 ambulances and responded to field emergencies across all provinces in South Korea. Inclusion criteria were: (1) employment as an EMT within the national 119 EMS system and (2) regular assignment to an ambulance crew with field response duties during the study period. EMTs meeting these criteria were eligible to participate regardless of current or past BWC use. Personnel not performing field ambulance duties (e.g., office-based roles) and incomplete or duplicate responses were excluded.

The survey was conducted using a self-administered online questionnaire from April 15 to May 15, 2024 (31 days). A survey link was distributed nationwide via the National Fire Agency’s internal electronic document system. Before accessing the questionnaire, potential participants were informed of the study’s purpose, procedures, voluntary nature of participation, anonymity, and right to withdraw at any time. Informed consent was provided electronically, and completion of the questionnaire was considered implied consent. To encourage participation, the National Fire Agency’s 119 Emergency Medical Services Division issued an official encouragement notice to all provincial fire departments nationwide during the final week of the survey period, requesting them to promote participation among their eligible EMTs. This institutional endorsement—from a higher authority than the survey-distributing institution (National Fire Research Institute)—is likely to have positively contributed to the response rate. The study protocol, including the waiver of written informed consent, was approved by the Institutional Review Board (IRB No. P01-202404-01-028).

Of 13,896 EMTs invited, 5,818 responded (response rate: 41.9%). After excluding questionnaires with missing key variables, 3,595 EMTs were included in descriptive analyses (61.8% of respondents; 25.9% of those invited). For analyses requiring complete workplace violence data (RQ1 and RQ3), 2,555 respondents with non-missing violence-related variables were analyzed (71.1% of the descriptive analysis sample; 43.9% of respondents; 8.4% of those invited).

Although the same dataset was previously used in a master’s thesis examining KAP-based behavioral constructs among EMTs^19^, the present study represents an independent analysis with distinct research questions, a different analytic framework, and a linkage to province-level assault indicators^16–18^. No previously published results overlap with the analyses presented in this manuscript.

### Representativeness of respondents

To assess the representativeness of the survey respondents, we compared key characteristics (sex, rank, and region) of the analytical sample with the national distribution of 119 EMTs reported in the 2023 EMS Statistical Yearbook published by the National Fire Agency of South Korea (reference year: 2022; total workforce: 13,896 EMTs), the most recent official source available at the time of the survey. Rank distribution closely mirrored the national workforce (firefighter/private: 33.8% vs. 34.9% nationally; senior firefighter + lieutenant: 60.6% vs. 59.0%; captain or above: 5.6% vs. 6.1%; all within ± 2% points). Female EMTs were modestly overrepresented in the sample (23.2% vs. 18.7% nationally, excluding substitute personnel [*n* = 199]), likely reflecting higher survey engagement among female respondents. Regional representation varied: respondents from Seoul (5.0% vs. 10.8% nationally) and the Jeolla area (10.9% vs. 16.2%) were underrepresented, while the Gyeongsang area (34.5% vs. 28.3%) and Jeju area (4.2% vs. 2.0%) were overrepresented. Age-group distribution was not reported in the national yearbook and could not be compared. Although selection bias due to voluntary participation cannot be completely ruled out, the overall sample reasonably reflects the national 119 EMT workforce in terms of rank distribution, with moderate regional and sex deviations as noted above.

### Regional assault indicators and data linkage

Province-level assault indicators were obtained from the annual National 119 EMS Statistical Yearbooks (2020–2024)^16^, publicly accessible via the National Fire Agency of South Korea official website (https://www.nfa.go.kr/nfa/releaseinformation/statisticalinformation/main/?boardId=bbs_0000000000000019&mode=view&cntId=63&category=&pageIdx=&searchCondition=&searchKeyword=). These indicators represent the officially-recorded count of assault incidents involving 119 EMTs in each province per year, as compiled by provincial fire departments and reported to the National Fire Agency. Assault incidents are defined in accordance with the National Fire Agency’s Standard Operating Procedure 405 (SOP 405: Assault Prevention and Response Procedures for EMTs), which encompasses both physical assaults and credible threats of violence against EMTs during field operations. These are administrative records—distinct from the self-reported workplace violence assessed in this survey—and reflect annual officially-reported incident counts per province. Perpetrator characteristics (e.g., sex, age, or relationship to the affected worker) are not included in these provincial statistics, which represents a limitation acknowledged in the Limitations section. Individual survey responses were linked to provincial indicators using the provincial administrative code recorded for each respondent, yielding a two-level data structure: individual respondents (level 1) nested within provinces (level 2, k = 19 administrative divisions). For each province, a five-year mean assault index (ASSAULT_5YR_MEAN) was calculated across 2020–2024 to reduce year-to-year variability. Provinces were then categorized into tertiles (low, moderate, high) based on the empirical distribution of this mean index across all 19 divisions (ASSAULT_LEVEL), allowing for potential non-linear associations and facilitating interpretation across strata without assuming a linear dose–response relationship. A continuous variable scaled per 10-unit increase (ASSAULT_5YR_MEAN_10) was additionally generated to assess dose–response relationships while retaining full distributional information.

### Measures

#### Primary outcomes

Three individual-level binary outcomes were defined:

1. Frequent workplace violence (VIOL_FREQ_HI3).

Workplace violence was defined as any act of aggression directed at EMTs by patients, caregivers, or bystanders during EMS calls in the past 12 months. The survey assessed four types of violence, each measured using a 5-point frequency scale (1 = never to 5 = very often): (1) verbal abuse (e.g., insults, threatening language, humiliating remarks); (2) physical threats (intimidating gestures or behavior without physical contact); (3) physical assault (hitting, pushing, kicking, or other physical contact); and (4) sexual harassment or sexual violence. All four items were self-reported by respondents based on their personal experience. A composite frequency index was calculated across these four items, and respondents scoring 3 (highest category) were classified as experiencing frequent workplace violence (VIOL_FREQ_HI3 = 1), whereas scores of 0–2 were considered low to moderate (VIOL_FREQ_HI3 = 0).

2. High perceived need for BWCs (BC_NEED_HI).

The perceived necessity of BWCs was assessed on a 5-point Likert scale (1 = very necessary to 5 = not necessary at all); lower scores indicated greater perceived need. Responses were dichotomized as high need (scores 1–2) versus neutral or low need (scores 3–5).

3. Actual BWC use (BC_WEAR_BIN).

EMTs indicated whether they typically wore a BWC during field EMS calls.

These three variables were the prespecified primary outcomes of the study. No additional secondary outcomes were examined.

### Covariates

Individual-level covariates included age group, sex, EMS career duration (< 3, 3–10, ≥ 10 years), daily EMS call volume (< 5, 5–<10, ≥ 10 runs/day), rank, and job position (EMT vs. ambulance driver). Regional assault burden was incorporated using either the categorical variable ASSAULT_LEVEL or the continuous variable ASSAULT_5YR_MEAN_10.

### Statistical analysis

Descriptive statistics were used to summarize demographic, work-related, violence-related, BWC-related, and regional variables. Categorical variables are presented as frequencies and percentages, while continuous regional indicators are presented as mean ± standard deviation. The distribution of continuous regional indicators was examined using histograms and normal probability (Q–Q) plots, including detrended plots, and evaluated for normality using the Shapiro–Wilk statistical test. Although some departures from normality were observed, normality was not required for multivariable logistic regression; therefore, the continuous regional indicator was retained as a predictor.

For each of the three research questions (RQ1–RQ3), multivariable logistic regression models were fitted, and adjusted odds ratios (ORs) with 95% confidence intervals (CIs) were reported. To evaluate the robustness of associations with regional assault burden, two model specifications were used per outcome. Model 1 (categorical) was specified as: logit[P(Yi = 1)] = β₀ + β₁·Age + β₂·Sex + β₃·Career + β₄·CallVolume + β₅·Rank + β₆·Position + β₇·ASSAULT_LEVEL + ε_i_. Model 2 (continuous) was specified as: logit[P(Yi = 1)] = β₀ + β₁·Age + β₂·Sex + β₃·Career + β₄·CallVolume + β₅·Rank + β₆·Position + β₇·ASSAULT_5YR_MEAN_10 + ε_i_. Yi denotes the binary outcome (VIOL_FREQ_HI3 for RQ1; BC_NEED_HI for RQ2; BC_WEAR_BIN for RQ3); for RQ3, VIOL_FREQ_HI3 and BC_NEED_HI were additionally included as predictors. Model 1 included individual covariates and the categorical regional indicator (ASSAULT_LEVEL; reference = moderate) to allow for potential nonlinearity and facilitate interpretation across strata. Model 2 included individual covariates and the continuous regional indicator (ASSAULT_5YR_MEAN_10) to assess the dose–response relationship while retaining full information. Model fit and calibration were evaluated using the − 2 log likelihood, pseudo-R² measures (Cox & Snell and Nagelkerke), and the Hosmer–Lemeshow goodness-of-fit test.

Because the survey targeted the entire national 119 EMT workforce during the study period, an a priori sample size calculation was not performed. Instead, the adequacy of the analytic sample for multivariable modeling was evaluated based on the number of outcome events relative to the number of model parameters and the precision of effect estimates (95% CIs). All statistical analyses were conducted using IBM SPSS Statistics version 29.0, and two-sided p-values < 0.05 were considered statistically significant. To address the two-level clustered data structure (individual respondents nested within provinces, k = 19), sensitivity analyses were conducted using Generalized Estimating Equations (GEE) with an exchangeable working correlation structure, specifying province as the clustering unit. GEE was preferred over multilevel logistic regression given the small number of clusters (k = 19), which can lead to unstable random-effect variance estimates. The same covariate sets as the primary logistic regression models were used, and cluster-robust standard errors were applied. GEE results are reported alongside primary findings in the Results section.

## Results

### Participant characteristics

Among 3,595 EMTs included in the descriptive analyses, 63.3% were in their 30s, and 20.1% were aged ≥ 40 years; 76.8% were male. Most respondents served as EMTs (73.9%), with the remainder working as ambulance drivers (26.1%). Approximately one-third had < 3 years of EMS career (34.7%), 47.5% had 3–10 years, and 17.8% had ≥ 10 years of experience. Daily EMS call volume was 5–<10 runs/day for 42.8% of respondents, < 5 runs/day for 30.8%, and ≥ 10 runs/day for 26.4%.

Nearly two-thirds of the respondents (64.4%) reported routine BWC use during EMS calls, and 87.9% reported a high perceived need for BWCs. Among the 2,555 respondents with complete workplace violence data, almost all (99.7%) reported at least one form of workplace violence in the past year, and 17.3% were classified as experiencing frequent violence. Based on the ASSAULT_5YR_MEAN, 19.1% of respondents were located in low-, 26.7% in moderate-, and 54.2% in high-assault regions (Table 1).

**Table 1:** General characteristics of participating 119 EMTs and key variables (*N* = 3595).

Variable	Category	*n*	%
Age group	20s	597	16.6
	30s	2274	63.3
	≥ 40s	724	20.1
Sex	Male	2761	76.8
	Female	834	23.2
Position	EMT (including assistant)	2657	73.9
	Ambulance driver	938	26.1
EMS career	< 3 years	1249	34.7
	3–10 years	1707	47.5
	≥ 10 years	639	17.8
Rank	Firefighter (Private)	1215	33.8
	Senior FF + Lieutenant	2180	60.6
	Captain or higher	200	5.6
Daily EMS call volume	< 5 runs/day	1108	30.8
	5–<10 runs/day	1539	42.8
	≥ 10 runs/day	948	26.4
Region	Seoul	181	5
	Capital area	858	23.9
	Gangwon area	256	7.1
	Chungcheong area	515	14.3
	Jeolla area	393	10.9
	Gyeongsang area	1242	34.5
	Jeju area	150	4.2
Workplace violence in past year	No (0)	8	0.3c
	Yes (1)	2547	99.7c
Frequent violence exposure	Low–moderate (0–2)	2113	82.7c
	Frequent (3)	442	17.3c
Body-worn camera use	No (0)	1280	35.6
	Yes (1)	2315	64.4
Perceived need for BWC	Low/neutral (0)	436	12.1
	High (1)	3159	87.9
Regional assault level	Low	687	19.1
	Moderate	961	26.7
	High	1947	54.2

Variable	*N*	Mean ± SD	Min–Max
ASSAULT_5YR_MEAN	3595	22.13 ± 24.34	1–74
ASSAULT_3YR_MEAN	3595	23.94 ± 27.12	1–80

Among 2,555 respondents with complete violence data. Workplace violence, BWC use, and perceived need for BWCs were all self-reported within a knowledge–attitude–practice (KAP) survey design.

### Frequent workplace violence (RQ1)

In the multivariate model, which included individual covariates and the ASSAULT_LEVEL (Table 2), several individual factors were associated with frequent workplace violence. Compared with EMTs in their 30s, those aged ≥ 40 years had lower odds of frequent violence (adjusted OR 0.66, 95% CI 0.46–0.93), whereas those in their 20s did not differ significantly. Female EMTs had lower odds than male EMTs (OR 0.65, 95% CI 0.50–0.85). EMTs with ≥ 10 years of experience had higher odds of frequent violence compared with those with 3–10 years of experience (OR 1.52, 95% CI 1.09–2.12). A higher daily call volume (≥ 10 vs. 5–<10 runs/day) was also associated with increased odds of frequent violence (OR 1.43, 95% CI 1.13–1.81). Ambulance drivers had lower odds of frequent violence than EMTs (OR 0.70, 95% CI 0.53–0.92).

**Table 2:** Factors associated with frequent workplace violence among 119 EMTs (RQ1, *N* = 2555).

Variable	Category	Total *n* (%)	Adjusted OR^a^	95% CI	*p*
Frequent violence exposure	Low–moderate (0–2)	2113 (82.7)	1.00 (ref)	–	–
	Frequent (3)	442 (17.3)	–	–	–
Age group	30s	1655 (64.8)	1.00 (ref)	–	–
	20s	408 (16.0)	0.78	0.56–1.10	0.159
	≥ 40s	492 (19.3)	0.66	0.46–0.93	0.019
Sex	Male	1907 (74.6)	1.00 (ref)	–	–
	Female	648 (25.4)	0.65	0.50–0.85	0.001
EMS career	3–10 years	1275 (49.9)	1.00 (ref)	–	–
	< 3 years	835 (32.7)	0.84	0.57–1.23	0.362
	≥ 10 years	445 (17.4)	1.52	1.09–2.12	0.015
Daily call volume	5–<10 runs/day	1166 (45.6)	1.00 (ref)	–	–
	< 5 runs/day	555 (21.7)	0.75	0.56–1.01	0.058
	≥ 10 runs/day	834 (32.6)	1.43	1.13–1.81	0.003
Rank	Senior FF / Lieutenant	1572 (61.5)	1.00 (ref)	–	–
	Firefighter (Private)	853 (33.4)	0.87	0.59–1.28	0.475
	Captain or higher	130 (5.1)	0.94	0.54–1.64	0.833
Position	EMT (incl. assistant)	1954 (76.5)	1.00 (ref)	–	–
	Ambulance driver	601 (23.5)	0.70	0.53–0.92	0.01
Regional assault level	Moderate	609 (23.8)	1.00 (ref)	–	–
	Low	471 (18.4)	1.13	0.81–1.59	0.471
	High	1475 (57.7)	1.22	0.93–1.60	0.144

^a^Adjusted odds ratios from multivariable logistic regression including all listed covariates; reference categories are indicated as “ref.” All outcomes are self-reported within a knowledge–attitude–practice (KAP) survey design.

In contrast, regional assault burden was not independently associated with frequent workplace violence. Compared with moderate-assault regions, both low- and high-assault regions showed adjusted ORs close to unity, with confidence intervals that included 1. Consistent findings were observed when regional assault burden was modeled as a continuous variable: the adjusted OR per 10-unit increase in ASSAULT_5YR_MEAN was 1.01 (95% CI 0.97–1.06), indicating no statistically significant association (Table 3). GEE sensitivity analysis confirmed these results, with all individual-level associations and the null finding for regional assault burden remaining substantively unchanged after adjustment for within-province clustering (Table 3).

**Table 3:** Association between 5-year mean regional assault index and three outcomes among 119 EMTs.

Outcome variable	Outcome *n* / *N* (%)	Primary logistic OR	95% CI	*p*	GEE OR	95% CI	*p*
Frequent workplace violence	442 / 2555 (17.3)	1.01	0.97–1.06	0.664	1.01	0.98–1.05	0.423
High perceived need for BWCs	3159 / 3595 (87.9)	0.96	0.92–1.01	0.104	0.97	0.91–1.04	0.434
BWC use during EMS calls	1683 / 2555 (65.9)	0.92	0.88–0.95	< 0.001	0.93	0.85–1.01	0.074

^a^Adjusted odds ratios were estimated from multivariable logistic regression models including the same individual-level covariates used in Tables 2 and 4, and 5. The predictor “5-year mean regional assault index (per 10-unit increase)” corresponds to the scaled variable *ASSAULT_5YR_MEAN_10* (ASSAULT_5YR_MEAN ÷ 10).

### Perceived need for BWCs (RQ2)

In the multivariable model for high perceived need for BWCs (Table 4), sex was a notable predictor: female EMTs had higher odds of reporting a high need compared with male EMTs (OR 1.60, 95% CI 1.21–2.11). A trend toward higher perceived need was observed among less experienced EMTs (< 3 years vs. 3–10 years; OR 1.36, 95% CI 0.95–1.95), although this did not reach statistical significance. Other individual characteristics, including age, daily call volume, rank, and job position, showed no consistent associations with perceived need.

**Table 4:** Factors associated with high perceived need for body-worn cameras (RQ2, *N* = 3595).

Variable	Category	Total *n* (%)	Adjusted OR^a^	95% CI	*p*
High perceived need for BWCs	Low/neutral (0)	436 (12.1)	1.00 (ref)	–	–
	High (1)	3159 (87.9)	–	–	–
Age group	30s	2274 (63.3)	1.00 (ref)	–	–
	20s	597 (16.6)	1.17	0.84–1.63	0.356
	≥ 40s	724 (20.1)	0.96	0.70–1.31	0.792
Sex	Male	2761 (76.8)	1.00 (ref)	–	–
	Female	834 (23.2)	1.60	1.21–2.11	< 0.001
EMS career	3–10 years	1707 (47.5)	1.00 (ref)	–	–
	< 3 years	1249 (34.7)	1.36	0.95–1.95	0.095
	≥ 10 years	639 (17.8)	1.23	0.88–1.71	0.220
Daily call volume	5–<10 runs/day	1539 (42.8)	1.00 (ref)	–	–
	< 5 runs/day	1108 (30.8)	0.91	0.72–1.15	0.427
	≥ 10 runs/day	948 (26.4)	1.13	0.87–1.47	0.348
Rank	Senior FF / Lieutenant	2180 (60.6)	1.00 (ref)	–	–
	Firefighter (Private)	1215 (33.8)	1.15	0.80–1.65	0.459
	Captain or higher	200 (5.6)	1.18	0.70–2.01	0.532
Position	EMT (incl. assistant)	2657 (73.9)	1.00 (ref)	–	–
	Ambulance driver	938 (26.1)	1.09	0.85–1.39	0.498
Regional assault level	Moderate	961 (26.7)	1.00 (ref)	–	–
	Low	687 (19.1)	1.14	0.84–1.55	0.391
	High	1947 (54.2)	1.03	0.81–1.31	0.821

^a^Adjusted odds ratios from multivariable logistic regression including all listed covariates; reference categories are indicated as “ref.” All outcomes are self-reported within a knowledge–attitude–practice (KAP) survey design.

Similar to findings for frequent workplace violence, regional assault burden was not significantly associated with perceived BWC need. Compared with moderate-assault regions, both low- and high-assault regions had adjusted ORs close to 1. When regional assault burden was modeled as a continuous variable, a 10-unit increase in ASSAULT_5YR_MEAN was associated with a non-significant decrease in the odds of high perceived need (OR 0.96, 95% CI 0.92–1.01; Table 3). GEE sensitivity analysis confirmed these null findings for regional assault burden. Notably, the GEE model additionally revealed a significant association between career duration of less than 3 years and higher perceived BWC need compared with 3–10 years (GEE OR 1.35, 95% CI 1.10–1.66, *p* = .004), consistent in direction and magnitude with the primary analysis (OR 1.36, *p* = .095) but reaching statistical significance after accounting for within-province clustering (Table 3).

### Actual BWC use (RQ3)

In the analysis of actual BWC use (Table 5), several individual- and violence-related factors were associated with camera use. Compared with EMTs in their 30s, those in their 20s had lower odds of using BWCs (OR 0.72, 95% CI 0.56–0.93), whereas EMTs aged ≥ 40 years did not differ significantly. Shorter EMS career (< 3 years vs. 3–10 years) was also associated with lower BWC use (OR 0.71, 95% CI 0.52–0.97). Interestingly, EMTs with lower daily call volume (< 5 vs. 5–<10 runs/day) had higher odds of BWC use (OR 1.31, 95% CI 1.03–1.66).

**Table 5:** Factors associated with body-worn camera use during EMS calls (RQ3, *N* = 2555).

Variable	Category	Total *n* (%)	Adjusted OR^a^	95% CI	*p*
BWC use	No (0)	872 (34.1)	1.00 (ref)	–	–
	Yes (1)	1683 (65.9)	–	–	–
Age group	30s	1655 (64.8)	1.00 (ref)	–	–
	20s	408 (16.0)	0.72	0.56–0.93	0.011
	≥ 40s	492 (19.3)	0.93	0.69–1.24	0.613
Sex	Male	1907 (74.6)	1.00 (ref)	–	–
	Female	648 (25.4)	0.84	0.68–1.04	0.108
EMS career	3–10 years	1275 (49.9)	1.00 (ref)	–	–
	< 3 years	835 (32.7)	0.71	0.52–0.97	0.032
	≥ 10 years	445 (17.4)	0.94	0.70–1.26	0.671
Daily call volume	5–<10 runs/day	1166 (45.6)	1.00 (ref)	–	–
	< 5 runs/day	555 (21.7)	1.31	1.03–1.66	0.025
	≥ 10 runs/day	834 (32.6)	1.11	0.91–1.35	0.318
Rank	Senior FF / Lieutenant	1572 (61.5)	1.00 (ref)	–	–
	Firefighter (Private)	853 (33.4)	1.00	0.73–1.38	0.979
	Captain or higher	130 (5.1)	0.75	0.48–1.17	0.201
Position	EMT (incl. assistant)	1954 (76.5)	1.00 (ref)	–	–
	Ambulance driver	601 (23.5)	0.41	0.33–0.51	< 0.001
Frequent violence exposure	Low–moderate (0–2)	2113 (82.7)	1.00 (ref)	–	–
	Frequent (3)	442 (17.3)	1.19	0.94–1.50	0.150
Perceived need for BWCs	0 = low/neutral	313 (12.3)	1.00 (ref)	–	–
	1 = high	2242 (87.7)	1.99	1.55–2.57	< 0.001
Regional assault level	Moderate	609 (23.8)	1.00 (ref)	–	–
	Low	471 (18.4)	0.32	0.24–0.43	< 0.001
	High	1475 (57.7)	0.26	0.21–0.34	< 0.001

^a^Adjusted odds ratios from multivariable logistic regression including all listed covariates; reference categories are indicated as “ref.” All outcomes are self-reported within a knowledge–attitude–practice (KAP) survey design.

Job position and perceived need were strongly associated with BWC use. Ambulance drivers were substantially less likely than EMTs to use BWCs (OR 0.41, 95% CI 0.33–0.51). EMTs with a high perceived need for BWCs had nearly twice the odds of using cameras compared with those with low or neutral need (OR 1.99, 95% CI 1.55–2.57). Frequent exposure to workplace violence (VIOL_FREQ_HI3) showed a positive but non-significant association with BWC use.

In contrast to the null findings for RQ1 and RQ2, regional assault burden was inversely associated with actual BWC use. Using the categorical measure (ASSAULT_LEVEL), EMTs in low-assault regions had lower odds of BWC use compared with moderate-assault regions (OR 0.32, 95% CI 0.24–0.43), and those in high-assault regions also had reduced odds (OR 0.26, 95% CI 0.21–0.34). When modeled as a continuous variable, a 10-unit increase in ASSAULT_5YR_MEAN was associated with lower odds of BWC use (OR 0.92, 95% CI 0.88–0.95; Table 3). GEE sensitivity analyses confirmed the inverse associations for the categorical measure (low vs. moderate: GEE OR 0.34, 95% CI 0.14–0.81, *p* = .015; high vs. moderate: GEE OR 0.29, 95% CI 0.15–0.57, *p* < .001), supporting the robustness of this finding after accounting for within-province clustering. However, the continuous regional measure became borderline non-significant after GEE adjustment (OR 0.93, 95% CI 0.85–1.01, *p* = .074), consistent with the expected attenuation when within-province correlation is properly accounted for, as the effective information contributed by a province-level variable is reduced relative to standard logistic regression.

## Discussion

In this nationwide survey of South Korean EMTs, workplace violence was nearly universal, with a substantial minority reporting frequent exposure over the past year. Despite this high burden, regional assault indicators derived from EMS statistics showed limited alignment with individual experiences of frequent violence. A critical consideration when interpreting this finding is the fundamental difference in measurement approaches between the two violence indicators. The survey-based measure captured self-reported personal experiences across four domains (verbal abuse, physical threats, physical assault, and sexual harassment) during the past 12 months, assessed through a KAP-based questionnaire. In contrast, the provincial assault indicators represent officially-recorded and administratively-compiled assault incidents derived from the national EMS dispatch reporting system under SOP 405, aggregated at the province level over multiple years. These differences—self-report versus administrative record, individual versus province-level aggregation, broader versus narrower operational definition of violence, and retrospective recall versus official documentation—likely contribute substantially to the observed disconnect between personal violence exposure and regional assault burden^1–5,16^. Individual-level factors such as age, sex, EMS experience, daily call volume, and job position were more strongly associated with frequent violence, consistent with previous research in EMS settings^1–5^. This aligns with recent evidence emphasizing that EMS workplace violence is multifactorial, shaped by a combination of situational, organizational, and individual determinants rather than by broad geographic measures alone^21–23^.

Perceived need for BWCs was very high overall, with nearly nine out of ten EMTs reporting that cameras were necessary. Female EMTs were more likely than males to report a higher perceived need, suggesting that sex differences in perceived vulnerability or safety may influence attitudes toward BWCs^7^. However, regional assault burden again did not meaningfully differentiate perceived need, indicating that EMTs’ desire for protective equipment may reflect broader concerns about safety in prehospital care rather than local assault statistics alone^1–5^. In line with recent studies describing diverse predictors and triggers of EMS workplace violence, these findings support the view that risk perceptions may be shaped by repeated exposure to high-risk scenarios and system-level stressors that are not fully captured by province-level indices^21,22^.

In contrast, actual BWC use was most strongly influenced by organizational role, perceived need, and, somewhat unexpectedly, regional assault burden. Ambulance drivers were substantially less likely than EMTs to wear BWCs, which may reflect differences in assigned equipment, job duties, or local policies. High perceived need was associated with nearly twice the odds of BWC use, highlighting that individual attitudes and organizational support may play important roles in translating policy into practice^8–11,14,15,19,20^. Interestingly, BWC use was lower in provinces with higher assault indices, even after adjustment for individual characteristics. This pattern may reflect more active implementation in regions with lower baseline assault burden or indicate that logistical, financial, or policy constraints limit camera deployment in the highest-burden areas^8–11,14,15^. These findings underscore that implementation decisions are shaped by organizational capacity, local governance, and operational priorities rather than need alone—an issue increasingly noted in contemporary EMS safety discussions^23,24^.

The lack of a clear association between regional assault burden and individual experiences of frequent violence suggests that province-level indicators may not adequately capture the micro-context of EMS encounters^1–5^. Assaults may be concentrated in specific urban neighborhoods, facility types, or call categories that are not reflected at the provincial level. Additionally, reporting practices and categorization in administrative statistics may differ from EMTs’ firsthand experiences^7,16^. From a policy perspective, these findings underscore the importance of integrating regional surveillance data with frontline reports when designing violence prevention strategies^1–6,8–11^. Recent research further emphasizes the need to consider incident- and context-level mechanisms of violence (e.g., scene characteristics, patient factors, and operational stressors) when developing interventions and interpreting aggregate statistics^21,22^.

This study has several strengths. To our knowledge, it is the first nationwide analysis linking individual EMT survey data to regional assault indicators within a unified EMS system^1–5,16^. The large sample size and use of multivariable models enabled adjustment for multiple individual-level confounders. Furthermore, we examined not only violence exposure but also perceived need for BWCs and actual BWC use, providing a comprehensive perspective on how BWCs fit into the EMS safety landscape and complementing prior literature^1–5,8–11,14,15,19,20^. By situating our findings alongside recent studies on predictors, triggers, and impacts of violence against EMS personnel, our research contributes to the growing body of evidence supporting multi-component approaches to workforce protection^21–24^.

However, this study has some limitations. First, the cross-sectional design precludes causal inference, making it impossible to determine whether BWC use reduces future violence or whether EMTs in more violent settings are more or less likely to adopt cameras^8–15^. Second, both violence exposure and BWC use were self-reported, which may introduce recall and social desirability biases. Third, regional assault indices were aggregated at the provincial level, potentially obscuring important local variations in risk^1–5,16^. Fourth, agency-level factors such as policies, training, or resource availability that could influence both BWC adoption and violence reporting, were not accounted for^8–11,14,15,19,20^. Fifth, approximately 29% of respondents had missing data on violence frequency, and analyses for RQ1 and RQ3 were limited to complete cases, which may introduce selection bias. Sixth, given the heterogeneity of violence mechanisms described in recent EMS research, unmeasured incident-level characteristics (e.g., call type, location context, and patient-related factors) may partially explain the limited correspondence between province-level indicators and individual experiences^21,22^. Seventh, province- or unit-level BWC policy data (e.g., mandatory versus discretionary use, equipment availability) were not systematically available for linkage to individual respondents, and potential policy-level confounding in BWC use models could not be controlled. Eighth, the provincial assault statistics do not include perpetrator characteristics (e.g., sex, age, or relationship to the affected worker), limiting the contextual depth of the regional indicators. Ninth, while GEE sensitivity analyses confirmed the robustness of most primary findings, the continuous regional assault measure became borderline non-significant after clustering adjustment for RQ3 (OR 0.93, *p* = .074), suggesting that conclusions regarding dose–response relationships between regional assault burden and BWC use should be interpreted with caution. Tenth, female respondents were modestly overrepresented relative to the national workforce (23.2% vs. 18.7%), and certain regions showed notable deviations from national proportions; although sex and region were controlled in all multivariable models, residual selection bias cannot be fully excluded.

Despite these limitations, our study highlights the high burden of workplace violence among South Korean EMTs and the widespread perceived need for BWCs, reflecting international concerns about violence against EMS providers and the search for effective protective strategies^1–6,8–11,14,15,19,20^. The findings also suggest that BWC implementation is influenced more by individual roles and organizational context than by regional assault burden alone. Policymakers and EMS leaders should consider integrating BWCs into comprehensive violence prevention strategies that include training, reporting systems, legal protections, and mental health support^1–3,6–11,14,15,19,20^. Recent research emphasizing both the determinants and broader occupational consequences of EMS workplace violence further supports the need for comprehensive, multi-level interventions rather than reliance on a single tool^21–24^. Prospective, multi-level studies are needed to evaluate the effectiveness and potential unintended consequences of BWCs in prehospital care, and to optimize their use as part of comprehensive workforce protection policies^3,8–15^. Such studies should ideally integrate individual survey responses with incident-level data and organizational policy information to clarify where, for whom, and under what conditions BWCs provide measurable benefit^22–24^.

## Conclusions

Workplace violence against EMTs is highly prevalent in South Korea, and the perceived need for BWCs is widespread. Individual characteristics, work conditions, and perceived need, rather than province-level assault burden, are key determinants of frequent violence and BWC-related behaviors. Notably, actual BWC use was lower in regions with higher recorded assault indices, suggesting that implementation does not always align with regional risk. Integrating BWCs into comprehensive EMS workforce protection strategies and tailoring implementation to local organizational contexts may enhance EMT safety in pre-hospital settings. Future research should employ prospective, multi-level designs linking incident-level EMS data (e.g., call type, scene, and patient factors) with organizational BWC policies, training, and resources to evaluate both effectiveness and potential unintended consequences. Such studies could clarify impacts on violence occurrence, reporting behavior, and EMT well-being, and identify implementation strategies that maximize benefits in high-risk settings.

## Data Availability

The individual-level survey data used and/or analyzed during the current study are not publicly available because of regulations governing operational EMS data and participant confidentiality in South Korea, but may be available from the corresponding author upon reasonable request and with permission from the National Fire Agency. The province-level assault statistics were obtained from the National 119 Emergency Medical Services (EMS) Statistical Yearbooks (2020–2024), published annually by the National Fire Agency of South Korea, which are publicly accessible at: https://www.nfa.go.kr/nfa/releaseinformation/statisticalinformation/main/?boardId=bbs_0000000000000019&mode=view&cntId=63&category=&pageIdx=&searchCondition=&searchKeyword=.

## Abbreviations

BWC: Body-worn camera
CI: Confidence interval
EMT: Emergency medical technician
EMS: Emergency medical service
OR: Odds ratio
VIOL_ANY: Any workplace violence in the past year
VIOL_FREQ_HI3: High-frequency workplace violence
BC_NEED_HI: High perceived need for body-worn cameras
BC_WEAR_BIN: Actual wearing of body-worn cameras
ASSAULT_LEVEL: Tertiles of five-year mean assault indicator
ASSAULT_5YR_MEAN: Five-year mean assault indicator
ASSAULT_3YR_MEAN: Three-year mean assault indicator
